# The outcome and parents-based cosmetic satisfaction following fixation of paediatric supracondylar humerus fractures treated by closed method with or without small medial incision

**DOI:** 10.1186/s40064-016-1846-9

**Published:** 2016-02-25

**Authors:** Serdar Hakan Basaran, Ersin Ercin, Alkan Bayrak, Mustafa Gokhan Bilgili, Cemal Kizilkaya, Uygar Dasar, Mustafa Cevdet Avkan

**Affiliations:** Department of Orthopaedics and Traumatology, Karabuk University Faculty of Medicine, Karabuk, Turkey; Orthopaedics and Traumatology Clinic, Bakırkoy Dr. Sadi Konuk Research and Training Hospital, Istanbul, Turkey

**Keywords:** Children, Supracondylar fracture, Percutaneous pinning, Cosmetic evaluation, Ulnar nerve injury

## Abstract

Supracondylar humerus fractures are common in children. Displaced fractures are usually treated with closed reduction and cross pin fixation. But, medial pinning may cause the ulnar nerve injury. The aim of this study was to compare the parents-based cosmetic satisfaction of the incision scars in children with displaced supracondylar humerus fractures treated by closed reduction and cross pin fixation with or without small medial incision. We retrospectively reviewed the medical records of 72 children with displaced supracondylar humerus fractures treated two different closed reduction and percutaneous pinning methods at our institution from January 2010 through December 2013. A group has 36 patients treated with small medial incision and crossed K-wires fixation after closed reduction. The other group has 36 patients treated with closed reduction and K-wires fixation. At the final follow-up, the patients were evaluated radiologically and clinically with Flynn’s criteria. Furthermore, a visual analogue scale was used to determine of the parents-based cosmetic satisfaction score. All fractures healed without major complications at the final clinical and radiological assessment. Although, between the two groups did not differ in terms of Flynn cosmetic and functional outcomes, there were statistically significant differences between both groups according to the parents-based cosmetic satisfaction scores. The closed reduction and crossed pin fixation without small medial incision should be preferred first because of better the parents-based cosmetic satisfaction.

## Background

The gold standard treatment of displaced supracondylar humerus fractures in children is the closed reduction and pin fixation (Pretell-Mazzini et al. 2011; Belhan et al. 2009; Sibinski et al. 2006; Kalenderer et al. 2008; Bashyal et al. 2009; Kaewpornsawan 2001). Cross pin fixation is more stable mechanically than any other type of pin configuration (Zionts et al. 1994; Lee et al. 2002). However, this fixation technique may cause iatrogenic ulnar nerve injury during the medial pinning. The probability of ulnar nerve injury in the fixation with crossed pins is higher than the fixation with only lateral entry pins (Brauer et al. 2007; Slobogean et al. 2010).

For eliminating iatrogenic ulnar nerve injury, some surgeons have preferred the fixation from only lateral side (Sibinski et al. 2006; Gaston et al. 2010). Moreover, many surgical techniques such as the fixation of fracture in the prone position (Fowler and Marsh 2006), medial pin placed without hyperflexion of the elbow (Eidelman et al. 2007; Shim and Lee 2002; Skaggs et al. 2001), ulnar nerve stimulation method (Michael and Stanislas 1996) and small medial incision over the medial epicondyle (Sibinski et al. 2006; Bashyal et al. 2009; Green et al. 2005; Khademolhosseini et al. 2013) have been described for reducing iatrogenic ulnar nerve injury. However, the medial mini-open technique causes extra scar formation.

The aim of this study was to compare the parents-based cosmetic satisfaction score of the incision scars in children with displaced supracondylar humerus fractures treated by closed reduction and crossed pin fixation with or without small medial incision. Also, we compared clinically and radiologically these treatment methods.

## Methods

A retrospective study was performed on patients with a displaced supracondylar fracture of the humerus treated from January 2010 to December 2013. Exclusion criteria were open fractures, fractures required open reduction, fractures with neurological or vascular injuries in admission, presence of any concomitant fractures, bilateral supracondylar humeral fracture, a previous ipsilateral elbow fracture, and loss to follow-up. We reviewed the hospital records in detail including personal data, preoperative clinical examinations, time to surgery, fracture type, time of pin removal and presence of complications.

**Fig. 1:**
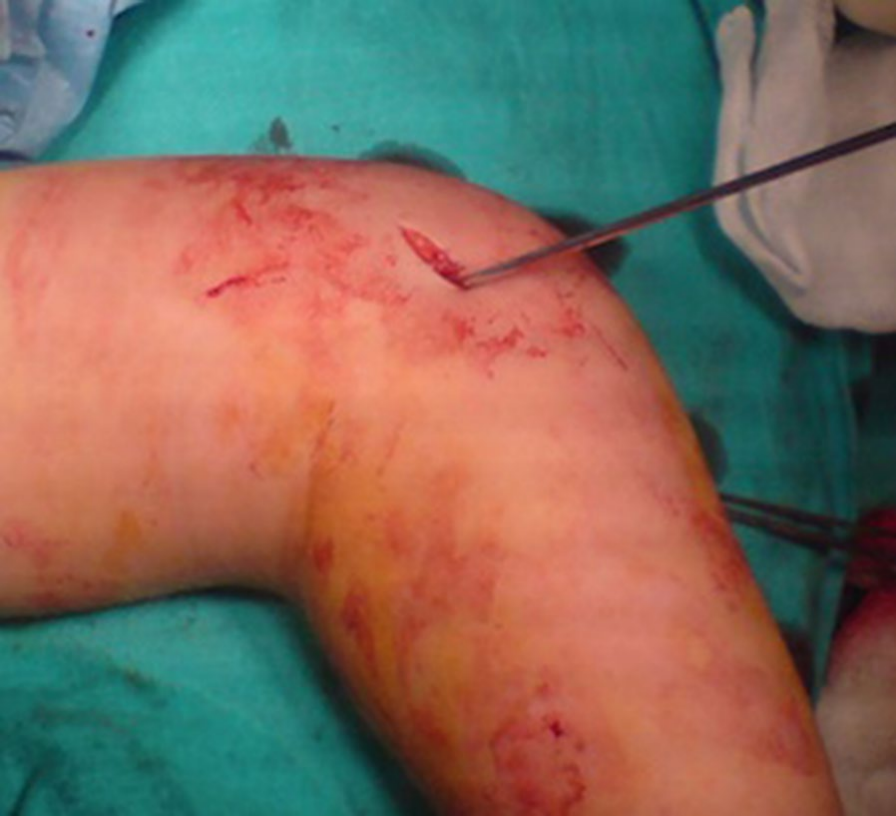
Small medial incision is seen

The patients were placed in supine position on the operating table. Closed reduction was performed under general anesthesia for all fractures. When reduction was maintained by manual pressure of the assistant, 1.8 or 2.0 mm Kirschner wires (K-wire) were inserted firstly from lateral epicondylar side and then were inserted the medial epicondylar side. Before the 1.8 or 2.0 mm K-wire was inserted from the medial side, medial epicondyle was palpated with thumb. Later, the thumb was shifted posteriorly to protect the ulnar nerve in group I, a approximately 10 mm small incision was also used to allow more safe pin placement over the medial epicondyle in group II (Fig. 1). We preferred cross-pin fixation with two or three K-wires. In both groups, the elbow was then extended to less than a 90° position to avoid injury to an anteriorly subluxating ulnar nerve before medial pin placement. The quality of reduction and fracture stability were examined intraoperatively both clinically and radiologically with the image intensifier. A long arm cast had been applied with approximately 70°–90° of elbow flexion and neutral forearm rotation for 3 weeks. After the cast was removed, ROM exercises were started while pins remained. The pins were removed after the determination of fracture healing. Later, active rehabilitation of the elbow was started.

The patients were evaluated clinically and radiologically at last follow-up visit. The clinical evaluation included assessment of the carrying angle, the passive range of elbow motion, scar formation, neurologic and vascular examinations of the fractured extremity, and determinations of any complications such as infection and the need for a reoperation. The radiographic evaluation included an anteroposterior radiograph of the distal part of the humerus and a lateral radiograph of the elbow. Baumann angle and humerocapitellar angle of fractured side and the differences of these angles between fractured and opposite sides were calculated and compared in both groups. Also, differences of carrying angle and passive range of elbow motion between fractured and opposite sides were calculated and were compared in both groups.

**Fig. 2:**
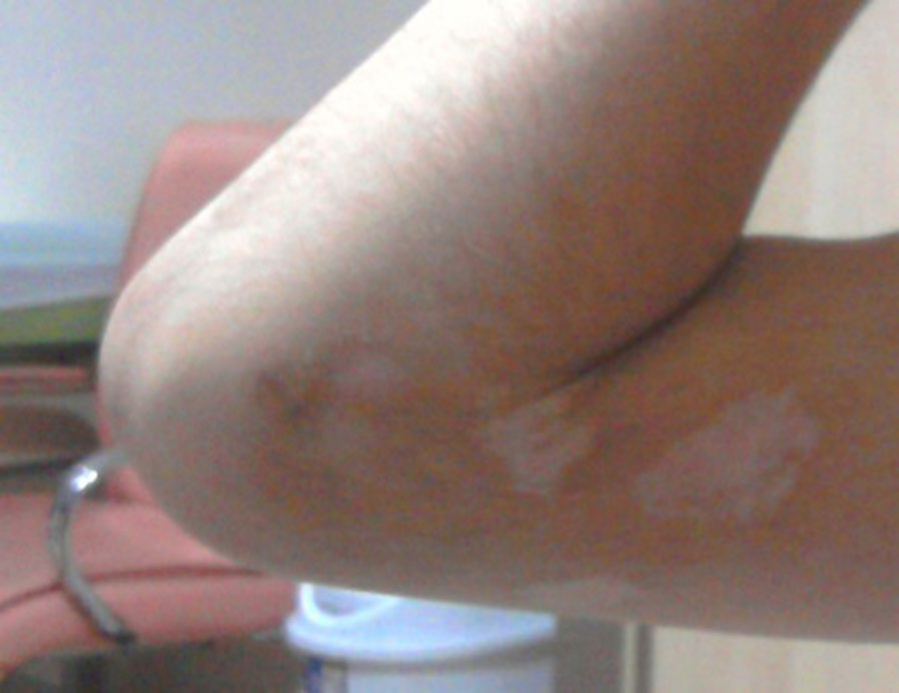
Scar appearance of the small medial incision of an 11 years old boy

At final follow-up, the patients were also evaluated as per the criteria of Flynn et al. (Flynn et al. 1974) For the parents-based cosmetic satisfaction evaluation of the scar formation was used a visual analogue scale (VAS) scoring by the families with ‘non-satisfied’ (at the 0-point end) and ‘most satisfied’ (at the 10-points end) (Fig. 2).

### Statistical analysis

IBM SPSS Statistic Version 20.0 software was used for statistical analysis. The data was evaluated with descriptive statistical methods (mean, standard deviation). Independent groups of quantitative data showing normal distribution was used independent samples test. For crude analysis of independent groups of qualitative data was used Chi Square test. A 95 % confidence interval, significance at p < 0.05 were accepted.

## Results

A total of 72 children fulfilled the inclusion criteria of the study, including 49 (68.1 %) boys and 23 (31.9 %) girls. Their mean age was 7.2 years (range 2–13 years); 22 children (30.6 %) had fractures on the right side and 50 (69.4 %) on the left side. All patients had Gartland type 3 fractures. Two study groups were set up from these included patients; the closed reduction percutaneous cross pin fixation was first group, and closed reduction percutaneous cross pin fixation with small medial incision was second group. The surgical methods were determined by the surgeons’ preferences.

The mean age of the patients was 6.9 years (range 2–13 years) in the group I and 7.4 years (range 2–13 years) in group II (p = 0.426). There were 22 males and 14 females (ratio 1.6:1, M:F) in group I and 27 males and nine females (ratio 3:1, M:F) in the group II (p = 0.312). The groups were statistically similar with regard to gender, age, follow-up time, time of the hospitalization, time to surgery and the pin removing time (Table 1).

**Table 1:** Relationship between both treatment groups

Characteristic	Group I (n = 36)	Group II (n = 36)	p value
Age (year)	6.9 ± 2.7	7.4 ± 2.9	0.426*
Gender (boy/girl)	22/14	27/9	0.312**
Time to surgey (min)	51.7 ± 21.8	59.7 ± 22	0.123*
Time of the hospitalization (day)	1.9 ± 1	2.1 ± 1.3	0.606*
Pin removing time (day)	30.2 ± 7.3	30.2 ± 4.5	0.969*
Follow-up (month)	23.3 ± 7.3	25.5 ± 11.4	0.340*

* Independent samples test

** Pearson Chi Square test

All patients healed completely in the final clinical and radiological assessments. None of the patients were seen major complications such as nerve/arterial injury, compartment syndrome, septic arthritis, osteomyelitis or nonunion. Also, none of the patients developed loss of reduction necessitating return to the operating room.

There were no significant differences in both groups the Baumann and the humerocapitellar angles at last follow up. Also differences of the Baumann angles, the lateral humerocapitellar angles, carrying angles and the elbow range of motion in between fractured and opposite sides were similar in both groups (Table 2). Excellent and good results of Flynn’s criteria were considered satisfactory. In 32 of the 36 patients were found satisfactory functional results in both groups. Moreover, all of the patients had satisfactory cosmetic results in both groups. There were no significant differences in terms of the cosmetic and functional outcomes in between two groups (p > 0.05). However, we found statistically significant differences between both groups as per the parents-based cosmetic satisfaction scores (Table 2).

**Table 2:** Radiological and clinical comparisons of two treatment groups

	Group I	Group II	p value
	Mean ± SS	Mean ± SS	
Baumann angle	19.6 ± 5.5	19.6 ± 7.5	0.986
Difference of Baumann angle	4 ± 4	4.6 ± 4.1	0.505
Humerocapitellar angle	41.9 ± 6.6	40.9 ± 8.1	0.548
Difference of humerocapitellar angle	4.6 ± 4.1	5.8 ± 5.6	0.296
Difference of carrying angle	2.3 ± 2.3	1.9 ± 2.0	0.360
Diffrence of range of elbow motion	4.5 ± 7.4	4.8 ± 8.1	0.880
VAS score	9.6 ± 0.8	8.6 ± 1.1	<0.001

Independent samples test

## Discussion

There are different surgical approaches can be used for displaced supracondylar humerus fractures in children (Ozkoc et al. 2004; Aktekin et al. 2008; Fu et al. 2011; Oh et al. 2003; Kazimoglu et al. 2009; Li et al. 2009; Basaran et al. 2015). Closed reduction and percutaneous pin fixation are a standard surgical treatment in these fractures (Pretell-Mazzini et al. 2011; Belhan et al. 2009; Sibinski et al. 2006). Also, the fixation with crossed pin is commonly preferred to provide greater rotational stability than lateral pin constructs (Lee et al. 2002; Brauer et al. 2007). However, use of the small medial incision causes extra scar formation. We compared mainly the parents-based cosmetic satisfaction scores of the incision scars of the two treatment methods in our study.

Open reduction is usually preferred after the unsuccessful closed reduction attempts. In the literature, both the cosmetic and functional outcomes based on Flynn’s criteria were similar in between closed reduction and open reduction performed through posterior (Ozkoc et al. 2004), anterior (Oh et al. 2003), lateral (Kaewpornsawan 2001) and medial (Fu et al. 2011) approaches. In these studies, cosmetic concerns are described by angular deformity of the upper extremity, whereas scar formation is not taken into consideration. Also, we did not encounter any studies evaluating cosmetically the medial mini-open method in the literature. Because the patients and their parents might be worried about the appearance of their skins, we think extra scar formation plays also an important role over the cosmetic outcomes.

We used VAS for the determination of parents-based cosmetic satisfaction score. The VAS is a quick and easy method of rating a subjective experience such as pain and anxiety (Oakley et al. 2009; Nicolas et al. 2010). Because our patients were children, we gave to the parents-based outcomes. The use of VAS gave to us an idea of how positive or negative about the experience that cosmetic satisfaction of the parents was. In our study, although the cosmetic results of Flynn criteria were similar, the parents-based cosmetic satisfaction score was better in closed reduction and crossed pins fixation without the small medial incision group.

The simplest way to avoid iatrogenic ulnar nerve injury is to not insert a medial pin. However, there is slightly probability of radial or anterior interosseous nerve damage associated with lateral pin fixation (Sibinski et al. 2006; Brauer et al. 2007). Different surgical techniques were used to prevent iatrogenic ulnar nerve injury (Sibinski et al. 2006; Bashyal et al. 2009; Brauer et al. 2007; Eidelman et al. 2007; Shim and Lee 2002; Skaggs et al. 2001; Michael and Stanislas 1996; Gordon et al. 2001; Wind et al. 2002; Shtarker et al. 2014). However, these techniques do not completely prevent ulnar nerve injuries (Brauer et al. 2007; Skaggs et al. 2001; Wind et al. 2002). The medial pin rarely impales directly the ulnar nerve. In addition, ulnar nerve palsy may develop due to entrapment by a stretched retinaculum after medial pin placement. In our opinion, after insertion of one or two lateral pins is temporarily sufficient to secure alignment, and <90° extension of the elbow relaxes the cubital tunnel retinaculum, a medial pin can be safely inserted to stabilize the fracture, and small medial incision was also performed by surgeons’ preference in some patients. In our study was not encountered any nerve palsies in both groups.

The loss of reduction is rarely encountered after crossed pins fixation and it usually occurs due to technical errors (Brauer et al. 2007; Omid et al. 2008). In present study, the reduction quality was same in both groups. Its retrospective nature was the main weak point of the current study. Another weak point was that the surgical techniques were selected by surgeons.

## Conclusion

Our study was showed both treatment techniques gave good results clinically and radiologically in treatment of supracondylar humerus fractures at the last follow up. However, the closed reduction and crossed pin fixation without small medial incision should be preferred first because of better the parent-based cosmetic satisfaction. If the ulnar nerve cannot be identified with palpation, a small incision can perform over the medial epicondyle to ensure protection of the ulnar nerve.
